# Adverse Effect of Nano-Silicon Dioxide on Lung Function of Rats with or without Ovalbumin Immunization

**DOI:** 10.1371/journal.pone.0017236

**Published:** 2011-02-17

**Authors:** Bing Han, Jing Guo, Tesfamariam Abrahaley, Longjuan Qin, Li Wang, Yuduo Zheng, Bing Li, Dandan Liu, Hanchao Yao, Jiwen Yang, Changming Li, Zhuge Xi, Xu Yang

**Affiliations:** 1 Laboratory of Environmental Sciences and Hubei Key Laboratory of Genetic Regulation and Integrative Biology, Huazhong Normal University, Wuhan, China; 2 Tianjin Institutes of Health and Environmental Medicine, Tianjin, China; 3 Division of Bioengineering, Nanyang Technology University, Singapore, Singapore; University of Leuven, Rega Institute, Belgium

## Abstract

**Background:**

The great advances of nanomaterials have brought out broad important applications, but their possible nanotoxicity and risks have not been fully understood. It is confirmed that exposure of environmental particulate matter (PM), especially ultrafine PM, are responsible for many lung function impairment and exacerbation of pre-existing lung diseases. However, the adverse effect of nanoparticles on allergic asthma is seldom investigated and the mechanism remains undefined. For the first time, this work investigates the relationship between allergic asthma and nanosized silicon dioxide (nano-SiO_2_).

**Methodology/Principal Findings:**

Ovalbumin (OVA)-treated and saline-treated control rats were daily intratracheally administered 0.1 ml of 0, 40 and 80 µg/ml nano-SiO_2_ solutions, respectively for 30 days. Increased nano-SiO_2_ exposure results in adverse changes on inspiratory and expiratory resistance (Ri and Re), but shows insignificant effect on rat lung dynamic compliance (Cldyn). Lung histological observation reveals obvious airway remodeling in 80 µg/ml nano-SiO_2_-introduced saline and OVA groups, but the latter is worse. Additionally, increased nano-SiO_2_ exposure also leads to more severe inflammation. With increasing nano-SiO_2_ exposure, IL-4 in lung homogenate increases and IFN-γ shows a reverse but insignificant change. Moreover, at a same nano-SiO_2_ exposure concentration, OVA-treated rats exhibit higher (significant) IL-4 and lower (not significant) IFN-γ compared with the saline-treated rats. The percentages of eosinophil display an unexpected result, in which higher exposure results lower eosinophil percentages.

**Conclusions/Significance:**

This was a preliminary study which for the first time involved the effect of nano-SiO_2_ to OVA induced rat asthma model. The results suggested that intratracheal administration of nano-SiO_2_ could lead to the airway hyperresponsiveness (AHR) and the airway remolding with or without OVA immunization. This occurrence may be due to the Th1/Th2 cytokine imbalance accelerated by the nano-SiO_2_ through increasing the tissue IL-4 production.

## Introduction

Environmental particulate matter (PM), especially the ultrafine PM, has been reported to be harmful to human health and exposure to PM from air pollution can lead to lung function impairment and exacerbation of pre-existing lung diseases, including chronic obstructive pulmonary disease (COPD) or asthma [1]–[3]. Diesel exhaust particles (DEPs) are the main component of PM and are characterized as a carbonic nucleus to absorb approximately 18,000 different high-molecular-weight organic compounds, of which the toxicities are known to be responsible for several diseases and inflammations of lung as well as many extrapulmonary syndromes [4]–[6]. Ambient PM is believed to promote lung inflammation through oxidative stress and lipid peroxidation [7]. Previous studies have demonstrated that DEPs can also exaggerate lipopolysaccharide-induced lung inflammation, representing an innate immunity-dominant lung inflammation [8], as well as OVA-induced lung inflammation, an adaptive immunity-dominant lung inflammation [9], [10].

In addition to conventional particles, recent innovations in nanotechnology have increased the availability of a new type of material described as nanoparticles [11], [12]. Nanoparticles are engineered structures with diameter ≤100 nm. They have been widely used in many fields, including medicine, microelectronics, photography, and pharmaceutical and cosmetic industries [11]–[13], but their possible hazards and risks have not been fully investigated to keep pace with their advances. Inhalation is the primary pathway through which we are exposed to nano-particles, the absorption of skin and ingestion of gut tract also contribute to the exposure of it [11]. Although *in vitro* and *in vivo* studies have indicated that nanoparticles can cause pulmonary and extrapulmonary toxicity [14]–[16], the mechanisms of these effects have not been extensively studied yet.

Asthma is a chronic lung disease with symptoms of obstruction of inhalation and exhalation caused by excess mucus production and swelling in airway membranes, leading to coughing and wheezing. Asthma cannot be completely healed or cured and thus needs continuous medical treatment, resulting in a large burden on society [17]. Allergic asthma is characterized by unbalance of Th1/Th2 cell and recruitment of type 2 T helper (Th2) cells. These cells release cytokines (e.g. IL-4, IL-5, and IL-13) to promote inflammatory cell influx, airway remodeling and AHR, while the production of type 1 T helper (Th1) cell's cytokines (e.g. IFN-γ and IL-2) is suppressed accordingly [18]–[20].

As a major component of the earth's crust, SiO_2_ has been known for its toxicity since ancient times [21], [22]. By virtue of its unique properties such as high specific surface area and adjustable pore size, nano-SiO_2_ has been recently employed in biosciences and medicines. Various silica nanoparticles have been used as drug vehicles or target-specific contrast agents for imaging [23]. Most previous studies have focused on conventional nano-SiO_2_ toxicities, such as silicosis, pulmonary tuberculosis, interstitial fibrosis, and emphysema, but the linkage between allergic asthma and silica dust exposure has not been studied [24]. Thus, the present study is designed in particular to evaluate the effect of nano-SiO_2_ on allergic asthma by exposing an OVA-treated rat to nano-SiO_2_-exposure solutions within intratracheally administered for 30 days.

## Materials and Methods

### Silica nanomaterials

**Figure 1:**
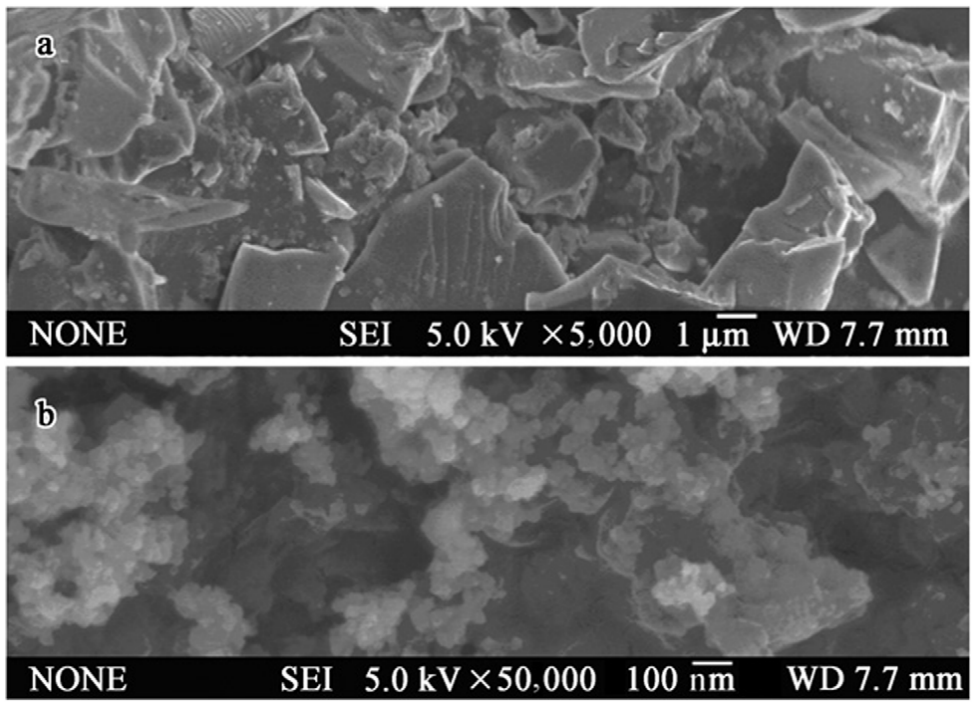
SEM images of nano-SiO_2_ (a) and nor-SiO_2_ (b). (JEOL-6700F).

Silica nanoparticles with diameters of 10–20 nm, 99.5% purity and BET surface area ranged from 140–180 m^2^/g were purchased from Sigma-Aldrich (USA). Scanning electron microscopy (SEM) images of the material were obtained by JEOL-6700F (Fig. 1) and compared with an SEM image of normal-sized SiO_2_ (nor-SiO_2_, 5–10 µm, Sinopharm, China) taken previously. The distilled water was used to prepare the mother solution (800 µg/ml). The prepared mother solution was sterilized at 120°C for 20 min to avoid the aggregation of nano-SiO_2_ induce by various microbes during our experimental period. The mother solution was daily stirred for 1 h followed by ultrasonicating for another hour to avoid clumping before each use. The exposure solutions (40 and 80 µg/ml) were freshly prepared by diluting the mother solution in distilled water and were ultrasonificated for another 15 min before its intretracheal instillation. After this process, both exposure solutions were stable for at least 24 h. Because the exposure solutions were freshly prepared before use, so sterilization for the two exposure solutions were not conducted.

### Animals and study protocol

**Figure 2:**
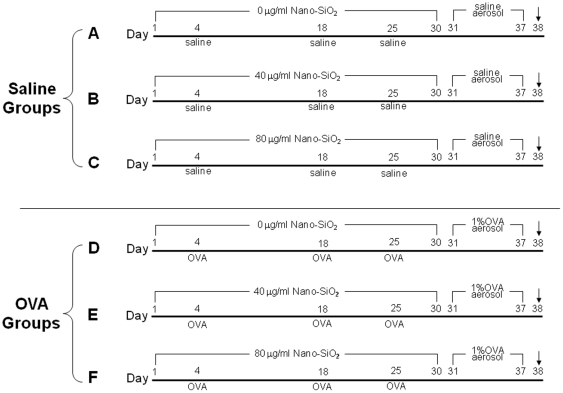
Study protocol. (A) Saline treatment plus 0 µg/ml nano-SiO_2_ exposure. (B) Saline treatment plus 40 µg/ml nano-SiO_2_ exposure. (C) Saline treatment plus 80 µg/ml nano-SiO_2_ exposure. (D) OVA treatment plus 0 µg/ml nano-SiO_2_ exposure. (E) OVA treatment plus 40 µg/ml nano-SiO_2_ exposure. (F) OVA treatment plus 80 µg/ml nano-SiO_2_ exposure. All rats were sacrificed on day 38.

Male Wistar rats (6–7 weeks old) were purchased from the Hubei experimental animal center (Wuhan, China), fed a commercial diet, and gave water ad libitum. The rats were housed in pathogen-free cages at 20–25°C and 50–70% relative humidity. The protocol of this study is shown in Figure 2. Rats were randomly divided into 6 experimental groups with 9 rats per group. The OVA-treated groups (group D, E and F) had OVA sensitization through subcutaneous injection of 1 ml of 200 µg/mL OVA solution containing 6.5 mg gelatinous Al(OH)_3_ (Sigma, USA) as an adjuvant at day 4, 18, and 25, followed by an aerosol challenge with 1% OVA for 30 min using an ultrasonic nebulizer (Yuyue, version 402AI, China) from days 31 to 37. Similar procedure was performed on the saline-treated groups (group A, B and C) in which OVA was replaced by saline. The exposure period lasted 30 d (from day 1 to 30), in which rats were instilled daily intratracheally with 0.1 ml of 1 of the 2 exposure solutions discussed above or saline. Finally, the rats were sacrificed at day 38 for further biochemical and histological analysis. This protocol was approved by the Office of Scientific Research Management of Huazhong Normal University with Certification on Application for the Use of Animals dated May 20^th^, 2007. All procedures strictly adhered to the guidelines from the National Committee of Animal Care and Use in the experiments.

### Intratracheal administration

Instillation of tested particles is usually employed in studies of particle toxicology [25]. To precisely control lung exposure to the tested material, the solutions were instilled intratracheally to the rats. Such a practice greatly reduced the rat suffering and concurrently achieved the experimental needs. In more detail, a rat had been forced to inhale ether vapor until it lost acupuncture response. The dosage and inhalation time of ether for each rat varied because different rat possess different physiological characteristic. Its body was then suspended ventral side outward on an incline by hooking its incisors on a small metal loop at the top of the incline and a light located above the rat's chest. The tongue of the rat was then gently pulled out with tweezers, allowing the airway to be clearly observed by light-penetrating its chest, and 0.1 ml of exposure solution or saline was instilled into the lung through a syringe-mounted stainless steel tube inserted into the airway.

### Lung function measurement

Airway hyper-responsiveness (AHR) assessment was conducted for 24 h after the final aerosol challenge by using the AniRes2005 lung function system (Bestlab, version 2.0, China) according to the manufacturer's instructions. After anesthetization by intraperitoneal injection of 1% pentobarbital sodium (Urchem, China), the respiration of the rat was maintained by a computer-controlled small animal ventilator connected to the rat through a tracheal cannula. The respiratory rate and the time ratio of expiration/inspiration were preset at 75/min and 1.5∶1, respectively. An injector needle was then inserted into the rat jugular vein, through which 0.025, 0.05, 0.1 and 0.2 mg/kg body weight methacholine (O-Acetyl-b-methylcholine chloride, MCH, Sigma, USA) were injected successively at a 5 min-intervals. After each injection, Ri, Re R-areas (the graphic area between the peak value and baseline) and the valley value of Cldyn [26] were recorded for further analysis.

### Bronchoalveolar lavage (BAL) and cell counting

Following measurement of AHR, the lung of each rat was lavaged *in situ* with 3 successive 1 ml volumes of saline instilled by syringe. After gentle pressing of the rat chest several times, BAL fluids were collected and combined. All recovery ratios of different rats were around 90%. Then, 1 ml of BAL fluid was centrifuged at 1000 rpm for 10 min at 4°C. The sediment was then resuspended in 1 ml of saline and the total cell content of 200 µl aliquots was counted by hemocytometer. Another 400 µl of the cell suspension was centrifuged again and resuspended in 400 µl of a mixed solution containing 20 µl 2% Eosin Y sodium solution (Amresco, high purity grade, USA), 20 µl of acetone and 360 µl of deionized water. After this process, only the eosinophils were available in the solution. The eosinophils were then counted by hemocytometer. The results expressed as percent of eosinophils in the total cell count ((eosinophils/total cells) ×100%).

### IL-4 and IFN-γ measurements

After BAL processing, the right lung was removed, and glass-made homogenizers were used for preparation of a 10% tissue homogenate in which the IL-4 and IFN-γ contents were assessed according to the manufacturers' instructions, by using commercial ELISA kits (IL-4 kit, Bender MedSystems, Germany; IFN-γ kit, Dakewe, China).

### Lung histological assay

After BAL processing, the left lung was harvested for histological assay. Tissue pretreatments and preparation of hematoxylin and eosin (H&E) stained slices were carried out as previously described [27] and all slices were examined under a microscope (Leica DM 4000B, Germany).

### Statistical analysis

Covariance analysis was performed with the AHR assessment data using the Statistical Product and Service Solutions (SPSS, version 13.0) to calculate F and p values. p<0.05 was considered as significant difference and p<0.01 was considered as extremely significant difference. Data from the other measurements were analyzed by using Origin software (version 8.0). p<0.05 was considered as significant difference and p<0.01 was considered as extremely significant difference. All data were reported as means ±SE.

## Results

### SEM image Comparison between nano-SiO_2_ and nor-SiO_2_

The SEM of nano-SiO_2_ particles in Fig. 1, b displays aggregated as clumps of nanoparticles, while that of nor-SiO_2_ particles in Fig. 1, a illustrates large slab-shaped forms.

### Airway hyper-responsiveness assessment

**Figure 3:**
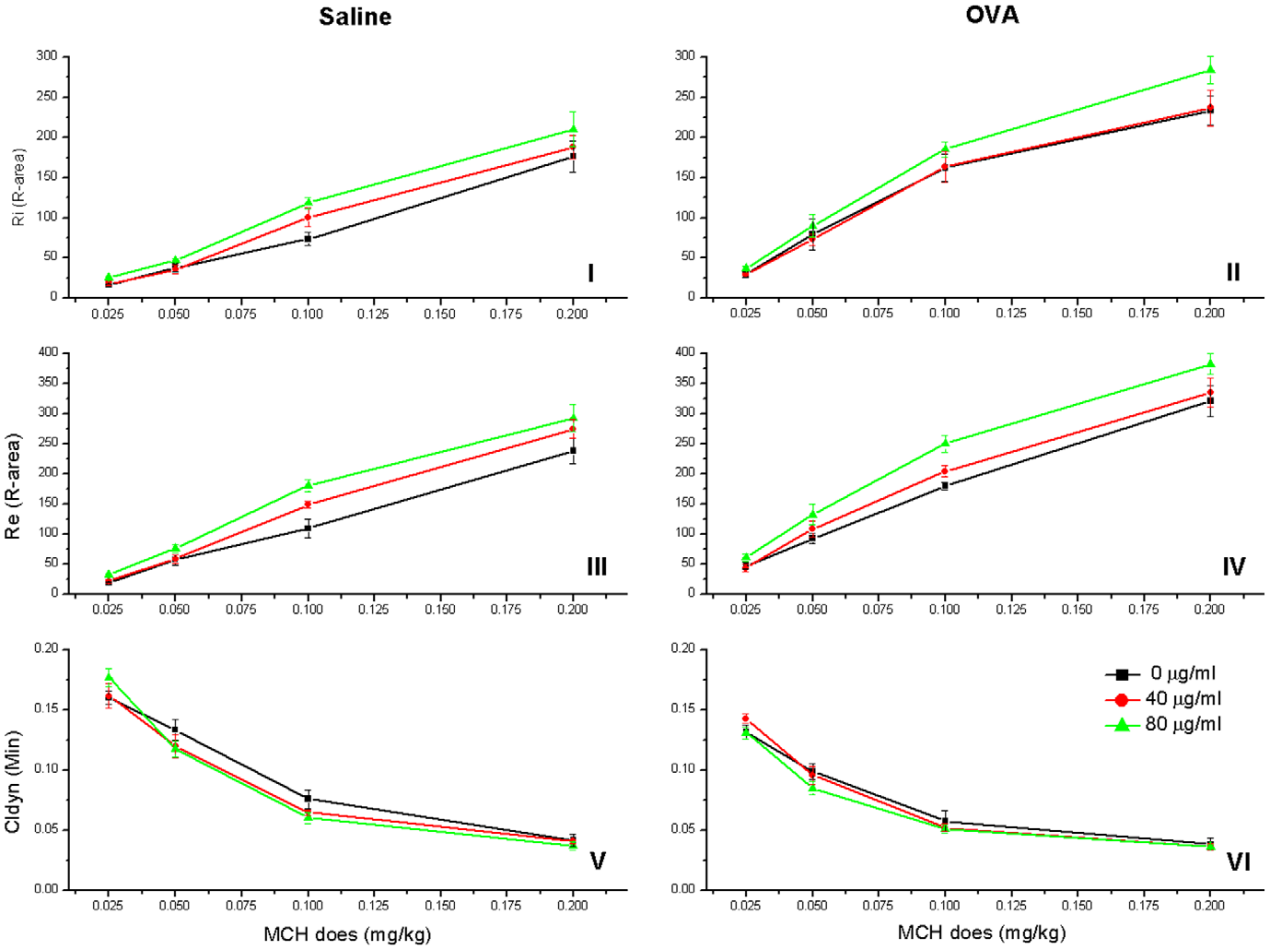
AHR assessment. I & II: Ri of saline groups and OVA groups, F_nanoSiO2_ = 6.460 (p = 0.002) and F_OVA_ = 64.898 (p = 0.000). III & IV: Re of saline groups and OVA groups, F_nanoSiO2_ = 19.059 (p = 0.000) and F_OVA_ = 83.118 (p = 0.000). V & VI: Cldyn of saline groups and OVA groups, F_nanoSiO2_ = 0.597 (p = 0.552) and F_OVA_ = 21.874 (p = 0.000).

Three parameters of the lung function (Ri, Re and Cydln) were recorded after each injection of MCH (0.025, 0.05, 0.1 and 0.2 mg/kg) (Fig. 3). Generally, Ri and Re values were higher in OVA groups (group D, E and F) in comparison to the saline groups (group A, B and C) (Ri, F = 64.898, p<0.01; Re, F = 83.118, p<0.01), and the higher exposure concentration of nano-SiO_2_ resulted in an upward shift in the Ri and Re curves (Ri, F = 6.460, p<0.01; Re, F = 19.059, p<0.01). A downward shift of the Cydln curves were detected as nano-SiO_2_ increased, but the different nano-SiO_2_ exposure concentrations appeared to have no significant effect (F = 0.597, p>0.05) on Cldyn. Moreover, the saline groups demonstrated higher Cydln values than the OVA groups (F = 21.874, p<0.01).

### Eosinophilic Percentage

**Figure 4:**
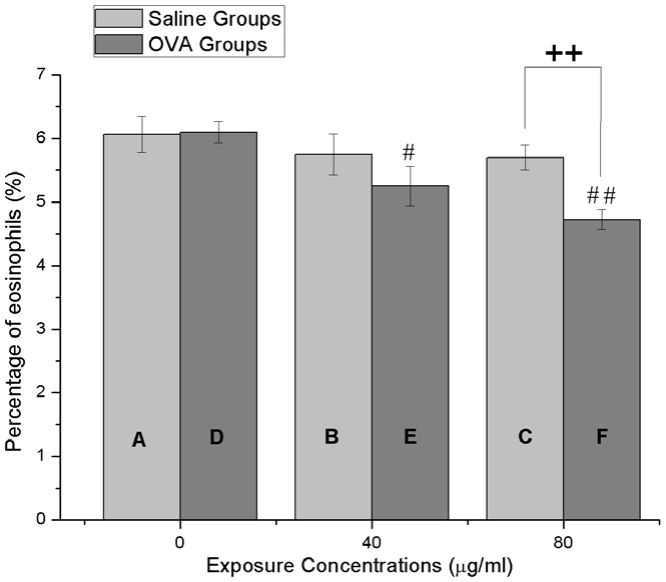
Eosinophil percentages in BAL fluids ((Eosinophils/total cells) ×100%). (A) Saline treatment plus 0 µg/ml nano-SiO_2_ exposure. (B) Saline treatment plus 40 µg/ml nano-SiO_2_ exposure. (C) Saline treatment plus 80 µg/ml nano-SiO_2_ exposure. (D) OVA treatment plus 0 µg/ml nano-SiO_2_ exposure. (E) OVA treatment plus 40 µg/ml nano-SiO_2_ exposure. (F) OVA treatment plus 80 µg/ml nano-SiO_2_ exposure. # p<0.05, ## p<0.01, all compared with (D). **++** p<0.01, comparisons between the same exposure concentration groups.

The eosinophil percentages appeared to indicate an unexpected tendency of that a dose-dependent decline existed in the OVA groups when the exposure concentration increased (Fig. 4). 40 µg/ml nano-SiO_2_-introduced OVA group (group E) demonstrated significant decrease of eosinophil percentage (p<0.05) when compared with the OVA-alone group without the introduced nanoparticles (group D), and 80 µg/ml nano-SiO_2_-introduced OVA group (group F) was extremely lower (p<0.01) compared with that of the OVA-alone group (group D). The eosinophil percentages of the saline groups also showed decreased tendency, but not significant. Furthermore, the eosinophil percentage in the 80 µg/ml nano-SiO_2_-introduced OVA group (group F) was extremely lower than that of 80 µg/ml nano-SiO_2_-introduced saline group (group C).

### IL-4 and IFN-γ measurement

**Figure 5:**
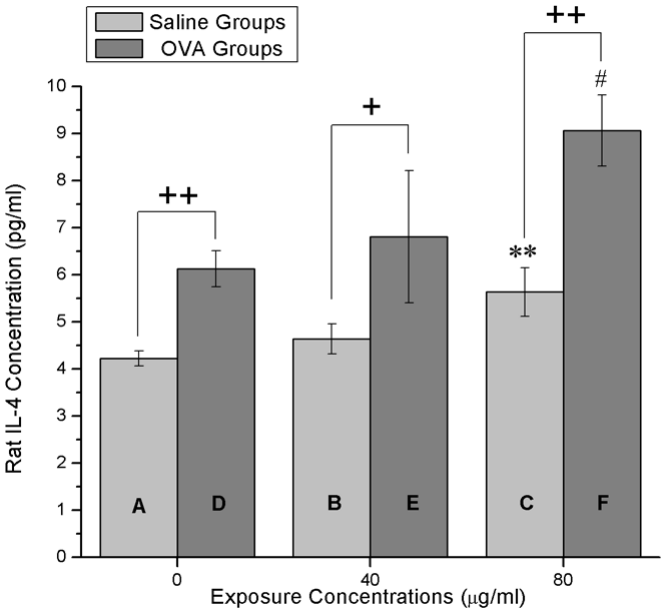
IL-4 concentrations. (A) Saline treatment plus 0 µg/ml nano-SiO_2_ exposure. (B) Saline treatment plus 40 µg/ml nano-SiO_2_ exposure. (C) Saline treatment plus 80 µg/ml nano-SiO_2_ exposure. (D) OVA treatment plus 0 µg/ml nano-SiO_2_ exposure. (E) OVA treatment plus 40 µg/ml nano-SiO_2_ exposure. (F) OVA treatment plus 80 µg/ml nano-SiO_2_ exposure. ** p<0.01, compared with (A). # p<0.05, compared with (D). **+** p<0.05, **++** p<0.01, comparisons between the same exposure concentration groups.

In saline groups or OVA groups, IL-4 content rose as the exposure concentration increased (Fig. 5). Moreover, the IL-4 content of the OVA groups was much higher than that in the saline groups (0 µg/ml: p<0.01, 40 µg/ml: p<0.05, 80 µg/ml: p<0.01) at a same nano-SiO_2_ exposure concentration. There was an extremely increase (p<0.01) in the 80 µg/ml nano-SiO_2_-introduced saline group (group C) when compared with the saline-alone group (group A). Significant increase (p<0.05) was also observed in the 80 µg/ml nano-SiO_2_-introduced OVA group (group F) in comparison to the OVA-alone group (group D).

**Figure 6:**
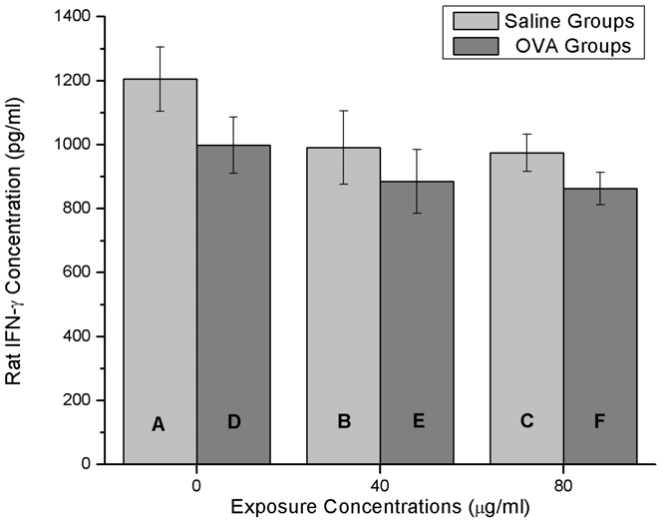
IFN-γ concentrations. (A) Saline treatment plus 0 µg/ml nano-SiO_2_ exposure. (B) Saline treatment plus 40 µg/ml nano-SiO_2_ exposure. (C) Saline treatment plus 80 µg/ml nano-SiO_2_ exposure. (D) OVA treatment plus 0 µg/ml nano-SiO_2_ exposure. (E) OVA treatment plus 40 µg/ml nano-SiO_2_ exposure. (F) OVA treatment plus 80 µg/ml nano-SiO_2_ exposure. No significant difference among groups.

A slight dose-dependent character of IFN-γ contents was observed in the 10% pulmonary homogenates (Fig. 6). In the saline groups or OVA groups, higher nano-SiO_2_ concentration led to lower, but not significant IFN-γ content. Generally, the saline groups (groups A, B and C) had higher IFN-γ contents than that in the OVA groups (groups D, E and F), but insignificantly.

### Lung histological assay

**Figure 7:**
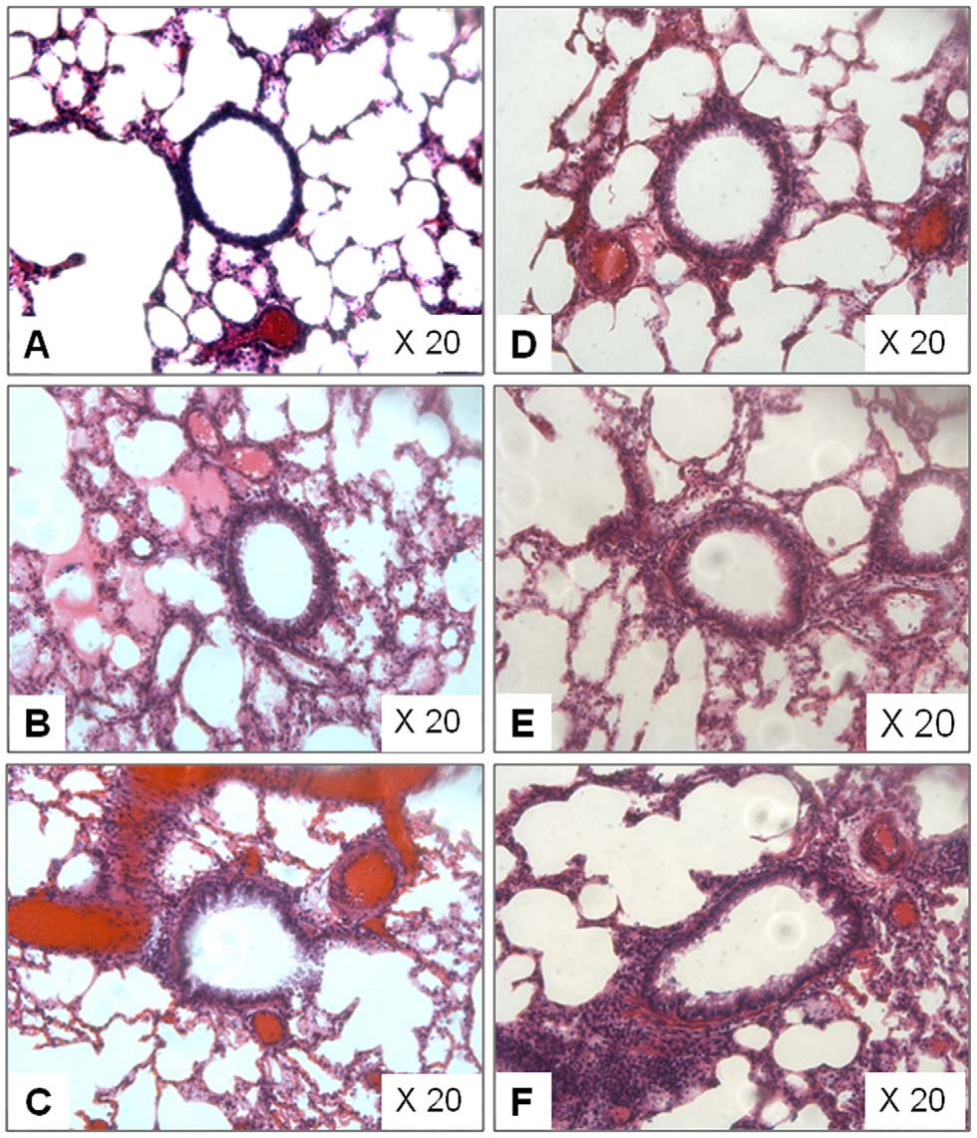
Representative histological images of H&E stained rats' lung. (A) Saline treatment plus 0 µg/ml nano-SiO_2_ exposure. (B) Saline treatment plus 40 µg/ml nano-SiO_2_ exposure. (C) Saline treatment plus 80 µg/ml nano-SiO_2_ exposure. (D) OVA treatment plus 0 µg/ml nano-SiO_2_ exposure. (E) OVA treatment plus 40 µg/ml nano-SiO_2_ exposure. (F) OVA treatment plus 80 µg/ml nano-SiO_2_ exposure. Slices microscopically examined at original magnification of 20× (Leica DM 4000B, Germany).

Representative images of the lung tissue slices in Figure 7 exhibits slices, possibly evidencing aggravated inflammation occurred in nano-SiO_2_-introduced saline groups (group B and C) and all OVA groups (group D, E and F). Obvious changes in the airway structures were observed in the 80 µg/ml nano-SiO_2_-introduced saline group (group C), and extensive airway remodeling appeared in the 80 µg/ml nano-SiO_2_-introduced OVA group (group F).

## Discussion

Unlike nor-SiO_2_, which possesses relatively larger sizes and sharper forms to cause possible physical cell damages (Fig. 1), the adverse effect of nano-SiO_2_ is most likely due to the nanoparticle biological toxicity. The diameter of silica nanoparticles used in this study ranged from 10–20 nm (Fig. 1) allows their easy access to the respiratory system and further introduce chemical or/and biological toxicity [11]. Respirable crystalline silica has been reported to be responsible for many diseases. Therefore, exposure limits during its use have been set by many organizations [24], [28], [29]. According to these limits and our research goal, the rats in this work were treated daily with intratracheal instillation of 0.1 ml of 0, 40, 80 µg/ml nano-SiO_2_ exposure solution, respectively. To simulate the real environmental exposure, 30 daily low dose instillations were administrated rather than few immediately high dose exposures before any allergen challenge.

The classic theory propounds that type I hypersensitivity is involved in the pathogenesis of allergic asthma, in which the AHR, airway remodeling and airway inflammation are 3 major characters of it [30], [18]. Bronchial hyperreactivity to pharmacological agents [18], one of the most important clinical discoveries in occupational asthma studies, was observed in our AHR assessment results (Fig. 3). The MCH challenge is a useful tool in diagnosis of allergic respiratory disorders and has been widely used in assessing airway responsiveness [31]. MCH as a pharmacological agent can cause increased Ri/Re and decreased Cydln. The inhalation, tail vein injection and jugular vein injection are three main administrations of MCH, but we found out that airborne MCH could coagulate before it enter the airway to jeopardize the accuracy of MCH challenge dose. Although the tail vein injection is an easy approach to conduct MCH challenge, the concentration of MCH in the blood is largely reduced due to a long travel from tail to lung. Consequently, after the pilot study, we chose the jugular vein injection for the MCH challenge, which could meet all the requests.

The R-areas of the respiratory resistances (Ri and Re) observed here explain the variation in the large airways, while the change of Cldyn represents the state of small airways or the parenchyma [26], [27]. The Ri and Re graphs (Fig. 3) show that increased nano-SiO_2_ exposure results in an upward shift of the curves (Ri/Re, p<0.01), suggesting that nano-SiO_2_ has an adverse effect on the large airways of the lung. A similar but reversed effect appears in the Cydln results, in which higher exposure causes a slight downward shift of the curves (p = 0.552), so the effects of nano-SiO_2_ on small airways or the parenchyma are not significant. According to these results, we propose that this is mainly because nano-SiO_2_ particles have been deposited in the large airways before reaching the small airways. Other studies have reported that ultrafine particles (<100 nm) can settle effectively in the alveolar region [12]. However, we believe that this should be attributed to the intratracheal instillation used in the present protocol, in which the nano-SiO_2_ particles (10–20 nm) are suspended in the liquid instead of air when entering into the airways.

The pulmonary histological assay (Fig. 7) evidences various degrees of inflammation appeared in all 6 cases. Considering the ether employed in the intratracheal instillation, we believe that the inflammations are partially caused by toxicity of ether. Additionally, diseases induced by SiO_2_ such as silicosis, industrial bronchitis, emphysema may also contribute to these inflammations. However, the higher nano-SiO_2_ exposure groups (both OVA groups and saline groups) appear to have more severe inflammation (inflammatory cell influx) than the lower groups, and generally more serious inflammation is found in the OVA groups compared with saline groups. Wall thickening, subepithelial fibrosis, mucous metaplasia, deposition of extracellular matrix in the subepithelial layer, myofibroblast hyperplasia, bronchial smooth muscle hyperplasia and hypertrophy, hyperplasia of submucosal glands, and increase of submucosal vessels have been defined as structural alterations to represent the remodeling manifestations of asthma [32], [33]. Based on these characteristics, obvious airway remodeling has been found in both 80 µg/ml nano-SiO_2_-introduced saline (group C) and OVA group (group F), but the latter has more severe degree of remodeling. Additionally, in the AHR assessment, Ri and Re volumes are generally higher in the OVA groups compared with the saline groups (Ri, F = 64.898, p<0.01; Re, F = 83.118, p<0.01; Fig. 4), and rats treated with saline shows increased Cydln volumes compared with OVA-treated rats (Cldyn, F = 21.874, p<0.01). Thus, both AHR assessment and pulmonary histological assay suggests that nano-SiO_2_ nanoparticle-treatment has stronger adverse effect on OVA rats than that on saline rats.

One of the fundamental features of allergic asthma has been thought to be the eosinophilic airway inflammation [18]. Therefore, eosinophilic percentage is chosen here as a major biomarker for allergic asthma, but our result indicates an unexpected dose-dependent pattern, in which the biomarker decreases rather than increases with increased nano-SiO_2_ exposure, especially in the OVA groups (Fig. 4). Unfortunately, the method used here is restricted to only the eosinophilic percentage. However, some studies have proposed that the typical eosinophilic pattern occurs in a minority of subjects and a noneosinophilic pattern is a distinct phenotype of asthma [34], [35]. Indeed, particulate air pollution has been thought to be a trigger for noneosinophilic airway inflammation in asthma [36]. Here, in light of the unexpected opposite pattern showed from the results, we speculate that nano-SiO_2_ may has been involved into the development and exacerbation of noneosinophilic asthma. However, further studies are needed to confirm this hypothesis with assessment of various inflammatory cells, cytokines and chemotactic molecules in BAL fluid.

Animal models for asthma have been used for over 100 years and considered as an ideal vehicle for testing and identifying the mechanisms behind the asthmatic phenotype. With appropriate methods, rat asthma models not only demonstrate increased responsiveness to nonspecific bronchoconstricting agents but also acute responses to allergen inhalation [37]. There are a plethora of approaches for building rat asthma models with different goals [38]–[42]. We have applied the protocol described above to create our own rat asthma model, in which more severe effects of AHR (Fig. 3) and airway remodeling (Fig. 7) can exist in the OVA-alone group (group D) compared with the saline-alone group (group A). Moreover, the OVA-alone group (group D) also possesses higher IL-4 content (Fig. 5) and lower IFN-γ content (Fig. 6) compared with the saline-alone group (group A). The eosinophilis percentages of group A and group D are not statistically different, and they all generally higher than those of other similar asthma models [39], [41]. However, before the administration of bronchoalveolar lavage, the lung function measurement, as a little surgery, might have created a mask effect and affected the eosinophilic percentages of the two groups (group A and D), respectively. Moreover, 30 daily consecutive intratracheal instillations we applied can also have the side effects even the exposure material is saline, which should also be considered. Nevertheless, the difference of inflammations between group A and group D can be clearly seen in H&E stained slices (Fig. 7A, D).

In animal asthmatic models, the Th1/Th2 cell balance is disturbed and Th2 cells are dominant in the airway. These cells induce IgE production and airway inflammation by producing Th2 cytokines such as IL-4, IL-5, and IL-13. The disturbance of the Th1/Th2 balance is also accompanied by decrease of Th1 cytokines such as IFN-γ and IL-2 [18]–[20]. Our experimental results show that the higher nano-SiO_2_ concentration induces rise of IL-4 content in the saline groups (group A, B and C) and the OVA groups (group D, E and F) (Fig. 5). Although the IFN-γ content barely declines after nano-SiO_2_ exposure in all groups (Fig. 6), we are convinced that nano-SiO_2_ could lead to a tissue IL-4 increase with or without OVA, which may, in turn, accelerate the Th1/Th2 cytokine imbalance and aggravate the symptoms of OVA-induced asthma such as AHR (Fig. 3) and airway remodeling (Fig. 7).

Immunological regulation is a very complex system where many things are still unknown. Nano-SiO_2_ may interact with one or some certain triggers located in the upstream of IL-4 generative pathway, and then promote the release of IL-4. According to the result of tissue IL-4 (Fig. 5), we can easily find that the IL-4 contents in the 80 µg/ml exposure groups (group C and F) are significantly higher than the control groups (group A and D), respectively. However, insignificant difference between 40 µg/ml exposure groups (group B and E) and control groups (group A and D) are also obvious, respectively. Therefore, we propose that there should be a threshold level of nano-SiO_2_ between 40 µg/ml and 80 µg/ml for IL-4 generating.

The relationship between SiO_2_ and the immune-mediated respiratory diseases, especially the allergic asthma, are seldom studied. This study demonstrates for the first time that intratracheal instillation of SiO_2_ nanoparticles could develop and exacerbate AHR and airway remolding with or without OVA immunization. This manifestation may attribute to the Th1/Th2 cytokine imbalance accelerated by nano-SiO_2_ through increasing the tissue IL-4 production. Additionally, nano-SiO_2_ nanoparticles may also involve in the development and exacerbation of noneosinophilic inflammation, which is definitely worthy of further study.
